# ActivinA Induced SMAD1/5 Signaling in an iPSC Derived EC Model of Fibrodysplasia Ossificans Progressiva (FOP) Can Be Rescued by the Drug Candidate Saracatinib

**DOI:** 10.1007/s12015-020-10103-9

**Published:** 2021-01-07

**Authors:** Susanne Hildebrandt, Branka Kampfrath, Kristin Fischer, Laura Hildebrand, Julia Haupt, Harald Stachelscheid, Petra Knaus

**Affiliations:** 1 Institute of Chemistry/Biochemistry, Thielallee 63, Freie Universität Berlin, 14195 Berlin, Germany; 2 Berlin-Brandenburg School for Regenerative Therapies (BSRT), Charité, Universitätsmedizin Berlin, Föhrer Str. 15, 13353 Berlin, Germany; 3 Charité - Universitätsmedizin Berlin, Augustenburger Platz 1, 13353 Berlin, Germany; 4 BIH Stem Cell Core Facility, Berlin Institute of Health (BIH), Anna-Louisa-Karsch-Straße 2, 10178 Berlin, Germany

**Keywords:** FOP, BMP-receptor, Activin, iPSCs, Human endothelial cells, HO, Saracatinib

## Abstract

Balanced signal transduction is crucial in tissue patterning, particularly in the vasculature. Heterotopic ossification (HO) is tightly linked to vascularization with increased vessel number in hereditary forms of HO, such as *Fibrodysplasia ossificans progressiva* (FOP). FOP is caused by mutations in the BMP type I receptor *ACVR1* leading to aberrant SMAD1/5 signaling in response to ActivinA. Whether observed vascular phenotype in human FOP lesions is connected to aberrant ActivinA signaling is unknown. Blocking of ActivinA prevents HO in FOP mice indicating a central role of the ligand in FOP. Here, we established a new FOP endothelial cell model generated from induced pluripotent stem cells (iECs) to study ActivinA signaling. FOP iECs recapitulate pathogenic ActivinA/SMAD1/5 signaling. Whole transcriptome analysis identified ActivinA mediated activation of the BMP/NOTCH pathway exclusively in FOP iECs, which was rescued to WT transcriptional levels by the drug candidate Saracatinib. We propose that ActivinA causes transcriptional pre-patterning of the FOP endothelium, which might contribute to differential vascularity in FOP lesions compared to non-hereditary HO.

**Supplementary Information:**

The online version contains supplementary material available at 10.1007/s12015-020-10103-9.

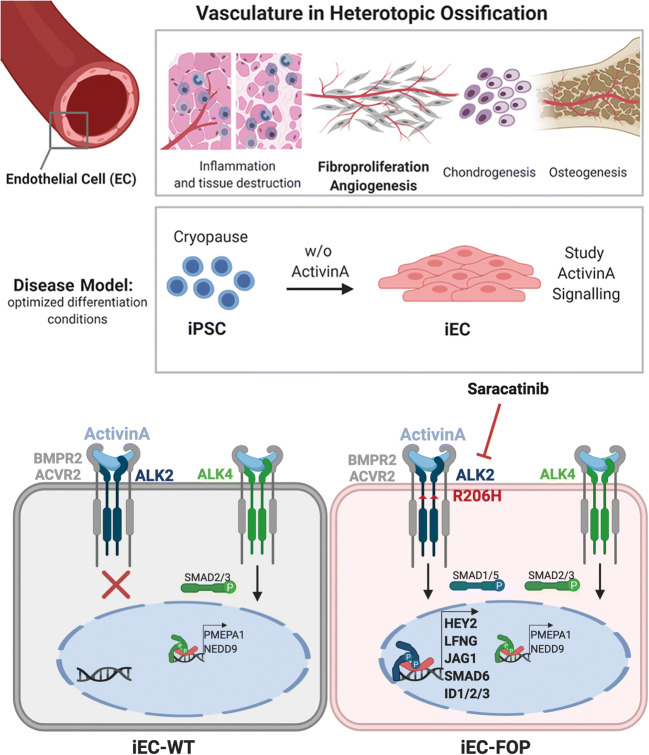

## Introduction

The vasculature is a complex, dynamic network of branching vessels lined by endothelial cells (ECs). Controlled blood vessel formation is crucial in embryogenesis and adult tissue homeostasis, requiring proper vascular patterning coordinated by multiple signaling cascades, including vascular endothelial growth factor (VEGF), NOTCH and Bone morphogenetic protein (BMP) pathways [1]. Aberrant activation of signaling pathways causes vascular malformations as observed in pathological conditions such as cancer and chronic inflammation [2]. The pathology of heterotopic ossification (HO) is defined by ectopic bone formation within soft tissues and is the main clinical symptom in rare hereditary forms of HO, but also a common issue of trauma and surgery [3]. Pre-osseous lesions are highly angiogenic and fibroproliferative, followed by an avascular chondrogenic stage and subsequent formation of mature, vascularized heterotopic bone through endochondral ossification [4–6]. Thus, blood vessels in HO undergo rapid and dynamic changes, but it remains elusive how signaling molecules orchestrate the vasculature in the aberrant tissue repair processes. It was recently shown that blockage of pro-angiogenic VEGFA reduced trauma-induced HO, highlighting vascularization as a therapeutic target [7].

Interestingly, human HO biopsies uncovered a differential vascular phenotype with increased vessel number, area and size in genetic versus non-hereditary forms distinguishing both pathologies [8]. *Fibrodysplasia ossificans progressiva* (FOP), a hereditary form of HO, is caused by gain of function mutations in the BMP type 1 receptor ACVR1 (ALK2) with R206H being the most common point mutation located in the intracellular glycine-serine (GS) rich domain [9]. Mutant receptors lead to hyperactivated SMAD1/5 signaling in response to BMPs [10] and aberrantly transduce SMAD1/5 signaling in response to ActivinA [11, 12]. Activins are TGF-β family members and normally signal via the type I receptors ACVR1B/C, intracellularly activating SMAD2/3 [13]. In FOP mice, blocking of ActivinA prevents HO indicating a central role of this ligand in the disease [11]. Whether ActivinA causes the vascular phenotype observed in human FOP biopsies is unknown.

ECs derived from induced pluripotent stem cells (iPSC), here called iECs, are an attractive in vitro model of the human endothelium. To date it is unclear whether iECs recapitulate primary ECs from FOP patients and are responsive to ActivinA. Current literature suggests that FOP iECs have reduced viability [14] and do not show aberrant ActivinA/SMAD1/5 signaling [15].

Here, we optimized iPSC differentiation conditions and generated a new iEC FOP model to investigate the effect of ActivinA on ACVR1 signaling in FOP iECs to better understand the underlying molecular mechanisms of the vascular phenotype. We demonstrate aberrant ActivinA/SMAD1/5 signaling and a unique transcriptome in FOP iECs interlinking ActivinA with BMP/NOTCH pathway activation. Moreover, we show that the drug candidate Saracatinib rescued the ActivinA-induced transcriptome in FOP iECs to WT levels suggesting a preventive effect on aberrant vascularization in early HO lesions in FOP.

## Materials and Methods

### iPSC Cell Culture

Cell lines iPSC-WT-1 (BCRTi005-A), iPSC-WT-2 (BCRTi004-A), iPSC-FOP-1 (BCRTi001-A), iPSC FOP-2 (BCRTi002-A) were generated from urinary cells as described previously [16–18]. All lines are registered, including ethical statements at the global hiPSC registry https://hpscreg.eu. iPSCs were cultured in defined conditions in E8 medium [19] in Geltrex-coated 6-well plates and routinely passaged at a ratio of ~1:20 every 4–5 days using 0.5 mM EDTA.

### Differentiation of iECs from iPSCs

Cryopreserved iPSCs (according to the CryoPause method in a controlled-rate freezer [20]), were thawed and immediately differentiated into iECs using a modified version of [21]. In brief, iPSCs were thawed in E8 medium supplemented with 10 μM ROCK inhibitor (Y-27632; Stem Cell Technologies, 72,305) and seeded at a density of 2–3 × 10^5^ cells/well on Geltrex coated 6-well plates. About 24 h after seeding the medium was changed to mesoderm induction medium containing N2B27 medium (1:1 mix of Neurobasal and DMEM/F12 with GlutaMAX and supplemented with N2 and B27 (−) Vitamin A (all Thermo Fisher), with 25 ng/mL BMP4 (PeproTech) and 6-7 μM CHIR99021 (BioVision). On day 4 the medium was changed to iEC induction medium consisting of StemPro-34 (Thermo Fisher) supplemented with 200 ng/mL VEGFA165 (PeproTech) and 2 μM Forskolin (Abcam). The iEC induction medium was renewed on day 5. On day 6 the EC fraction was measured by FACS CD144-FITC and sorted by MACS using CD144 MicroBeads (Miltenyi Biotec). Upon collection of the negative fraction, the positive fraction (CD144+) was eluted. Cells were resuspended in Expansion Medium [22] (EGM-2 containing single quots, w/o Hydrocortisone (LONZA) supplemented with 20% FCS, 100 units/ml penicillin, 10 μg/ml streptomycin (PAA-laboratories), 10 μM SB431542 (Selleck Chemicals)) and 4 × 10^4^ cells per cm^2^ were seeded on 0.1% gelatine (EmbryoMax, Sigma-Aldrich) coated culture flasks. Medium change was performed the next day and subsequently every 2–3 days. Cells were split (max. 1:3) or frozen (90% FCS, 10% DMSO) upon confluency. Upon thawing iECs were cultured in Growth Medium (EGM2 containing single quots, w/o Hydrocortisone (PromoCell) supplemented with 20% FCS, 100 units/ml penicillin, 10 μg/ml streptomycin) on 0.1% gelatin coated culture flasks and used for all experiments in passage 2.

### Cell Stimulation with Growth Factors and SMKI Treatment

Cells were starved prior growth factor stimulation for 4 h. iPSC were starved in Essential 6 medium (Thermo Fisher) and iECs in Endothelial Basal Medium 2 (EBM2) (PromoCell) supplemented with 0.5% FCS and 100 units/ml penicillin, 10 μg/ml streptomycin (PAA Laboratories). Small-molecule kinase inhibitors (SMKI) were added to cells 1 h prior ligand stimulation with indicated concentrations unless stated otherwise. Growth factors and SMKIs were reconstituted and stored according to manufacturer instructions. hBMP6 (S. Vukicevic, University of Zagreb, Croatia), rhActivin-A (R&D Systems), hTNF-α (ImmunoTools), Saracatinib, K02288, SB431542 (Selleck Chemicals).

### Adhesion Molecule Expression Assay

iECs were exposed to tumor necrosis factor-alpha (TNF-α) (0.6 nM) for 2 h. RNA was isolated and expression of *ICAM-1* was analyzed by qPCR.

### Barrier Function

Endothelial barrier function was assessed by Electric Cell-substrate Impedance Sensing (ECIS) using the ECIS Zθ instrument (Applied BioPhysics, ibidi). iECs were grown to confluence on 0.1% gelatine (EmbryoMax, Sigma Aldrich) coated 8W10E arrays (ibidi) (3 × 10 ^4^ cells/ well) for 72 h. Cells were starved for 5 h before stimulation with VEGFA (5 nM) and PBS as control. Barrier function was assessed by ECIS measurement of resistance at 4000 Hz.

### Tube Formation Assay

iECs were seeded in growth medium on growth factor reduced Matrigel (Corning) coated 96 well culture plates (3 × 10^4^/well). After an incubation for 24 h at 37 °C in 5% CO_2_ images were taken using phase-contrast microscopy.

### Western Blot Analysis

Protein lysates were separated on 10% SDS-PAGE gels. Gels were transferred to PVDF membranes by Western blotting. Membranes were blocked in 0.1% TBS-T containing 3% *w*/*v* BSA for 1 h at RT, washed three times in 0.1% TBS- T and incubated with indicated primary antibodies (GAPDH #2118; phospho-SMAD1/5 (Ser463/465) #9516; SMAD1 #6944; phospho-SMAD2 (Ser465/467) #3108, SMAD2 #3122, Cell Signaling Technology) overnight at 4 °C following manufacturer’s instructions. Secondary HRP conjugated antibodies goat-anti-mouse or goat-anti-rabbit IgG (Dianova) were used. Chemiluminescent reactions were processed using WesternBright Quantum HRP substrate (advansta) and documented by using a ChemiSmart5000 digital imaging system (Vilber-Lourmat).

### qRT-PCR

Cells were washed once with DPBS and RNA was isolated using NucleoSpin RNA II (Macherey-Nagel) according to manufacturer instructions. The amount of 0.5–1 μg RNA was reverse transcribed into cDNA using M-MLV reverse transcriptase and random primers (NEB). qRT-PCR was performed using PCR Luna Universal qPCR Master Mix (NEB) and specific primers listed in Table 1. Expression levels were assessed by StepOne Plus, and StepOne Software 2.3 (Applied Biosystems) and measured in technical replicates. Target gene expression was quantified relative to the housekeeping gene *RSP9* using the ΔΔCT method including primer efficiency [23].

**Table 1:** qRT-Primers

Human Gene	Human Primer name	Primer Sequences (5′ → 3′)
*RSP9*	RSP9 forward RSP9 reverse	CTGCTGACGCTTGATGAGAA CAGCTTCATCTTGCCCTCAT
*ID1*	ID1 forward ID1 reverse	GCTGCTCTACGACATGAACG GCTGCTCTACGACATGAACG
*ID2*	ID2 forward ID2 reverse	GTGGCTGAATAAGCGGTGTT TGTCCTCCTTGTGAAATGGTT
*ID3*	ID3 forward ID3 reverse	CTTCCGGCAGGAGAGGTT AAAGGAGCTTTTGCCACTGA
*SMAD6*	SMAD6 forward SMAD6 reverse	TGATGAGGGAGTTGGTACCC ACCTCCCTACTCTCGGCTGT
*ACVRL1*	ACVRL1 forward ACVRL1 reverse	ACAACATCCTAGGCTTCATCGC GGTTTGCCCTGTGTACCG
*ACVR1*	ACVR1 forward ACVR1 reverse	AAGCCTGGAGCATTGGTAA TCACTGGGGTACTCGGAGA
*BMPR1A*	BMPR1A forward BMPR1A reverse	CATCTTGGAGGAGTCGTAAGAA TTCTGTCCTTGAACACGAGAAA
*BMPR1B*	BMPR1B forward BMPR1B reverse	CTGCCATAAGTGAGAAGCAAAC ACAACGCAAGACCTTTGGAC
*ACVR1B*	*ACVR1B* forward *ACVR1B* reverse	TGCAACAGGATCGACTTGAG ATGATGCCTACCAGCTCCAC
*ACVR1C*	ACVR1C forward ACVR1C reverse	ACTTGTGCCATAGCGGACTTA GGTTCCCACTTTAGGATTCTGAG
*TGFBR1*	TGFBR1 forward TGFBR1 reverse	ACTGTAAAGTCATCACCTGGC GTGAATGACAGTGCGGTTGT
*BMPR2*	BMPR2 forward BMPR2 reverse	CATGGAGATGCGTAGCTGTC GGTTCTGAGGAAGTGCGAGT
*ACVR2A*	ACVR2A forward ACVR2A reverse	CCTGACAGCTTGCATTGCTGACTT TCTGCGTCGTGATCCCAACATTCT
*ACVR2B*	ACVR2B forward ACVR2B reverse	TGAAGCACGAGAACCTGCTACAGT GGCATACATGTCAATGCGCAGGAA
*TGFBR2*	TGFBR2 forward TGFBR2 reverse	GTTCAGAAGTCGGATGTGGAA TCTGGTTGTCACAGGTGGAA
*INHBA*	INHBA forward INHBA reverse	CCTCCCAAAGGATGTACCCAA CTCTATCTCCACATACCCGTTCT
*NOG*	NOG forward NOG reverse	GCGAGATCAAAGGGCTAGAG TAACTTCCTCCGCAGCTTCT
*KDR*	KDR forward KDR reverse	AGCGATGGCCTCTTCTGTAA ACACGACTCCATGTTGGTCA
*CDH5*	CDH5 forward CDH5 reverse	CAGCCCAAAGTGTGTGAGAA CGGTCAAACTGCCCATACTT
*PECAM1*	PECAM1 forward PECAM1 reverse	GAGTCCTGCTGACCCTTCTG TCAGGTTCTTCCCATTTTGC
*ICAM1*	ICAM1 forward ICAM1 reverse	CAAGGCCTCAGTCAGTGTGA CCTCTGGCTTCGTCAGAATC
*vWF*	vWF forward vWF reverse	ACTCATGGGCTCTGAGCAGT GCTCTTCAGAAGCTGGCACT
*VEGFR1*	VEGFR1 forward VEGFR1 reverse	GTTCAAGGAACCTCGGACAA GCTCACACTGCTCATCCAAA
*NRP1*	NRP1 forward NRP1 reverse	GCCTGCAACTTGGGAAACTGG CCTTGGTTGGATGATGTGATCTGG
*ENG*	ENG forward ENG reverse	ATGAGGCGGTGGTCAATATC AGGAAGTGTGGGCTGAGGTA
*NEDD9*	NEDD9 forward NEDD9 reverse	ATGGCAAGGGCCTTATATGACA TTCTGCTCTATGACGGTCAGG
*PMEPA1*	PMEPA1 forward PMEPA1 reverse	TGTCAGGCAACGGAATCCC CAGGTACGGATAGGTGGGC
*UNC5B*	UNC5B forward UNC5B reverse	GGTTTCCACCCCGTCAACTT GGGGATTTTGTCGGTGGAGT
*SGK1*	SGK1 forward SGK1 reverse	AGGATGGGTCTGAACGACTTT GCCCTTTCCGATCACTTTCAAG
*SMAD9*	SMAD9 forward SMAD9 reverse	GTTCACCACGGCTTTGAAGT TGACATCCTGGCGATGATAC
*HEY2*	HEY2 forward HEY2 reverse	TTGAAGATGCTTCAGGCAACAGGG TCAGGTACCGCGCAACTTCTGTTA
*JAG1*	JAG1 forward JAG1 reverse	GGGAACCCGATCAAGGAAATCAC CAGCAAGGGAACAAGGAAATCTGT
*LFNG*	LFNG forward LFNG reverse	CTGCACCATCGGCTACATCG GGCGTTCCGCTTGTTTTCAA

### Immunofluorescence Staining

3 × 10^4^ cells were seeded on glass coverslips placed in 24-well plates until they formed a confluent monolayer. Cells were fixed with 4% paraformaldehyde (PFA), quenched in 50 mM ammonium chloride and permeabilized in 0.5% Triton-X-100 for 15 min. After blocking for 1 h in 3% *w*/*v* BSA and 5% *v*/v normal goat serum in PBS, cells were stained with primary antibodies (VE-Cadherin, #2500, 1:400; PECAM-1 #3528, 1:200; Cell Signaling Technology) in blocking solution overnight. Fluorophore conjugated secondary antibodies, (1:300) (Alexa Fluor™ 488 F(ab’)2-goat-α-mouse IgG (A-10684, Invitrogen) and Alexa Fluor™ 594 goat-α-rabbit IgG (A-11012, Invitrogen)), were diluted in blocking solution and incubated for 1 h at RT. Nuclei were stained with DAPI (Sigma-Aldrich) using a 1:1000 dilution in DPBS for 30 min at RT.

### FACS Analysis

Antibodies for surface marker staining (CD144-FITC-human clone REA199, CD31-APC-human clone AC128 (Miltenyi Biotec)) were used according manufactures instructions. Measurement was performed by MACSQuant VYB (Miltenyi Biotec) and analyzed by FCS Express V6 software.

### RNA-Seq Library Preparation and Sequencing

8 × 10^4^ iECs per well were seeded in 12 well plate and grown to confluence generated from 4 biological independent iPSC lines. Two independent experiments of ligand and SMKI treatment were performed for each line. Upon starvation, ligand stimulation and SMKI treatment cells were lysed and RNA was isolated according to manufacturer instructions (Macherey-Nagel). RNA samples were sent for Sequencing to Genewiz, Leipzig, Germany.

### RNA-Seq Data Analysis

The sequencing data was uploaded to the Galaxy web platform, and the public server at *usegalaxy.eu* was used to analyze the data [24]. Quality of raw reads was performed with FASTQC before data was mapped to the reference genome (hg38) using STAR mapper [25]. Alignment quality was assessed with MultiQC [26] and RNA-Seq alignments were assembled into potential transcripts by StringTie [27]. This output was used to analyze differential gene expression with DESeq2 [28]. Cutoff for differentially expressed genes was a logarithmic fold change of ≥0.58 and with an adjusted *p* value of 0.05. Shared differentially expressed genes in both FOP donors was assessed with BioVenn [29]. Z-score calculation and generation of heatmaps was performed with the „pheatmap “package in RStudio. Gene ontology was performed with DAVID Bioinformatic Resources 6.8 [30, 31] and applying Benjamini correction.

### Statistical Analysis

All experiments of each of the four biological replicates (iPSC donors) was independently repeated at least three times. Statistical analysis and data illustration was performed with GraphPad Prism 8 (GraphPad Software Inc.). Normal distribution of data sets *n* < 5 were tested with the Shapiro-Wilk normality test. Data sets *n* ≥ 5 were tested additionally with the Kolmogorov Smirnov test for normality. In cases of failure to reject the null hypothesis, the ANOVA and Bonferroni post hoc test were used to check for statistical significance under the normality assumption. *P*-values lower than 0.05 were considered statistically significant (*P < 0.05, **P < 0.01, ***P < 0.001, ****P < 0.0001).

## Results

### Optimized Differentiation Conditions to Generate Functional ECs from FOP iPSCs

**Fig. 1:**
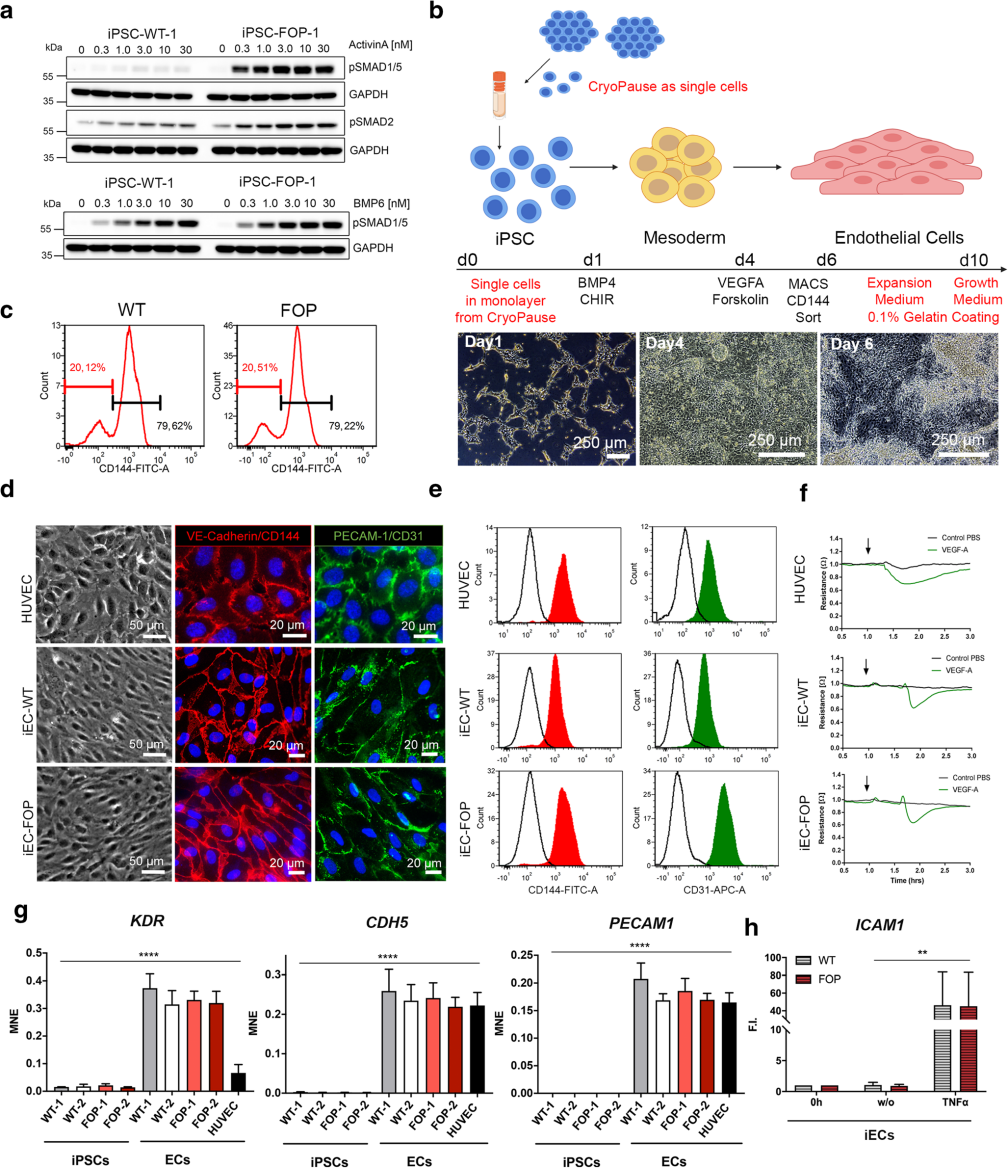
**Generation of FOP iECs from iPSCs.** (**a**) Representative Western blot of iPSCs after stimulation with different doses of ActivinA, BMP6 for 30 min (**b**) Workflow of iEC differentiation [21] with modification in red, created with BioRender.com. Below, phase contrast images of cells at different stages. (**c**) iEC differentation efficiency by FACS of CD144+ cells (WT-1; FOP1) at day 6. (**d**) Phase contrast and immunofluorescence stainings of EC markers CD144 and CD31 (iEC WT-1, FOP-1, HUVEC). (E) Representative FACS of CD144 and CD31 (WT-1, FOP-1, HUVEC). (F) Impedance measurement of VEGFA (2 nM) induced permeability of iEC and HUVEC monolayers. (G) RT-PCR of EC markers in iPSCs compared to iECs. Data is represented as mean normalized expression (MNE) ± SD, ****p < 0.0001 using one-way ANOVA (H) RT-PCR of *ICAM1* expression upon 2 h TNFα (0.6 nM) treatment in iECs. Data is shown as mean fold induction (F.I.) ± SD ** *p* < 0.01 using two-way ANOVA

In this study we aimed to investigate ActivinA signaling in FOP ECs. Due to a high risk of HO-induction in FOP patients by biopsy retrieval, the establishment of cell models has been challenging. iPSCs from two FOP patients (ACVR1 R206H) and two controls, which we characterized previously [16–18], were used to generate iECs (FOP-1, FOP-2, WT-1, WT-2) (Fig. S1a). All four iPSC lines responded to ActivinA with a dose-dependent increase in SMAD2 phosphorylation (pSMAD2) (Fig. 1a and S1b), while ActivinA-induced SMAD1/5 phosphorylation (pSMAD1/5) was only seen in FOP iPSCs (Fig. 1a and S1b). As a control, treatment with BMP6, an ACVR1 ligand, showed dose-dependent phosphorylation of SMAD1/5 in both, WT and FOP iPSCs (Fig. 1a and S1b). Based on aberrant ActivinA/SMAD1/5 signaling in FOP iPSCs, we optimized an EC differentiation protocol devoid of exogenous ActivinA [21] in combination with *CryoPause* [20] (Fig. 1b) by directly seeding iPSCs for differentiation after thawing. On day 1, mesoderm was induced by BMP4 and the GSK3-β inhibitor CHIR. On day 4, endothelial expansion of mesodermal cells was achieved by VEGFA and Forskolin. The EC fraction was measured by FACS and subsequently purified by MACS using vascular endothelial cadherin (VE-Cadherin/CD144) as a marker. WT and FOP iPSC differentiated to iECs with efficiencies up to ~80% (Fig. 1c). Both iECs form dense monolayers, express the junctional markers VE-Cadherin and PECAM-1 as assessed by immunofluorescence staining and FACS analysis (Fig.1d, e) and quantitative RT-PCR (Fig. 1g). iECs expressed additional EC markers, such as Vascular endothelial growth factor 1 and 2 (VEGFR1, VEGFR2), von Willebrand factor (vWF), Neuropillin (NRP1) and Endoglin (ENG) (Fig. 1g and S1c). To test the functionality of WT and FOP iECs in vitro, we show the formation of vessel like networks in a tube formation assay (Fig. S1d) and validate the formation of a tight endothelial barrier by impedance measurements, depicted as constant resistance values (Fig. 1f). Treatment with VEGFA caused decreased resistance with a subsequent recovery in iECs, similar to HUVECs (Fig. 1f). Moreover, endothelial response to pro-inflammatory TNF-α with a pro-adhesive phenotype was confirmed by expression of intracellular adhesion molecule-1 (ICAM-1) in iECs (Fig. 1h). In summary, WT and FOP iPSCs followed distinct ActivinA/SMAD responses and differentiated to ECs with high efficiencies without differences in the here assessed EC characteristics.

### FOP iECs Show a SMAD1/5 Response to ActivinA

**Fig. 2:**
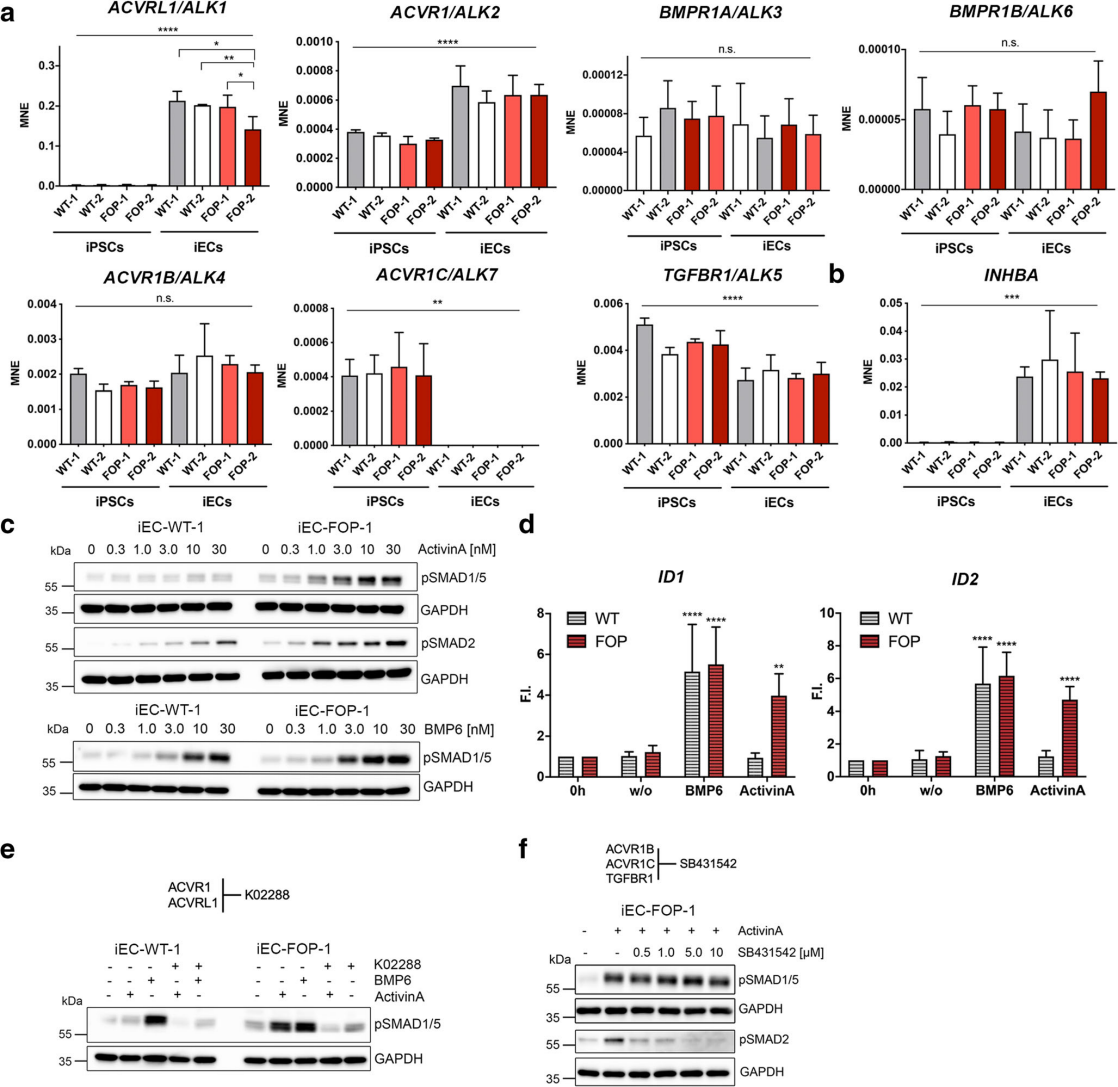
**Only FOP iECs gain SMAD1/5 responsiveness to ActivinA.** (**a**) RT-PCR of type I receptors in iPSCs compared to iECs. Data is shown as MNE ± SD. (**b**) RT-PCR of *INHBA* in iPSCs compared to iECs. Data is shown as MNE ± SD. (**c**) Representative Western blot of iECs after stimulation with different doses of ActivinA, BMP6 for 30 min. (**d**) RT-PCR of BMP target genes upon 2 h BMP6 (5 nM), ActivinA (5 nM) treatment in iECs. Data is shown as mean F.I. ± SD. (E-F) Representative Western blot of iECs pretreated with type I receptor inhibitors (**e**) K02288 (0.5 μM) and treatment with BMP6, ActivinA for 30 min. (**f**) SB431542 (dose series) and treatment with ActivinA for 30 min. (MNE; mean normalized expression), (F.I.; fold induction), * *p* < 0.05,** p < 0.01, ***p < 0.001, ****p < 0.0001 significance was calculated using one-way (A,B) and two-way ANOVA (D, relative to unstimulated (w/o))

Interestingly, *ACVR1* and *ACVRL1* where the only BMP/TGFβ receptors upregulated during EC differentiation of iPSCs with no differences between WT and FOP (Fig. 2a and S2a). ActivinA (*INHBA*) expression was highly increased in WT and FOP iECs compared to iPSCs (Fig. 2b). Transcriptomics revealed that among the Activin/BMP ligands, *INHBA* and *BMP6* showed the highest expression in iECs (Fig. S2b). While ActivinA increased pSMAD2 levels in WT and FOP iECs, strong SMAD1/5 phosphorylation was only seen in FOP iECs (Fig. 2c and S2c). ActivinA increased pSMAD1/5 levels dose- and time-dependently (Fig. 2c, Fig. S2c and S2d). In contrast, BMP6 induced phosphorylation of SMAD1/5 in WT and FOP iECs in a similar dose-dependent manner with slightly higher sensitivity of FOP iECs at 3 nM and 10 nM BMP6, indicating hypersensitive signaling (Fig. 2c and S2c).

Aberrant ActivinA signaling was confirmed on SMAD1/5 target gene expression (*ID1/2/3)* in FOP iECs only, while BMP6 induced expression of *ID1/2/3* in WT and FOP iECs (Fig. 2d and S2e)*.* To verify the contribution of type I receptors to ActivinA/SMAD1/5 signaling, we pretreated iECs with K02288, a specific ACVR1/ACVRL1 inhibitor (Fig. 2e) or SB431542 (ACVR1B/C/TGFBR1 inhibitor) (Fig. 2f) and show prevention of SMAD1/5 phosphorylation only by K02288, confirming ActivinA signaling through ACVR1.

### RNA-Seq Analysis Reveals a FOP-Specific Transcriptome upon ActivinA Stimulation

**Fig. 3:**
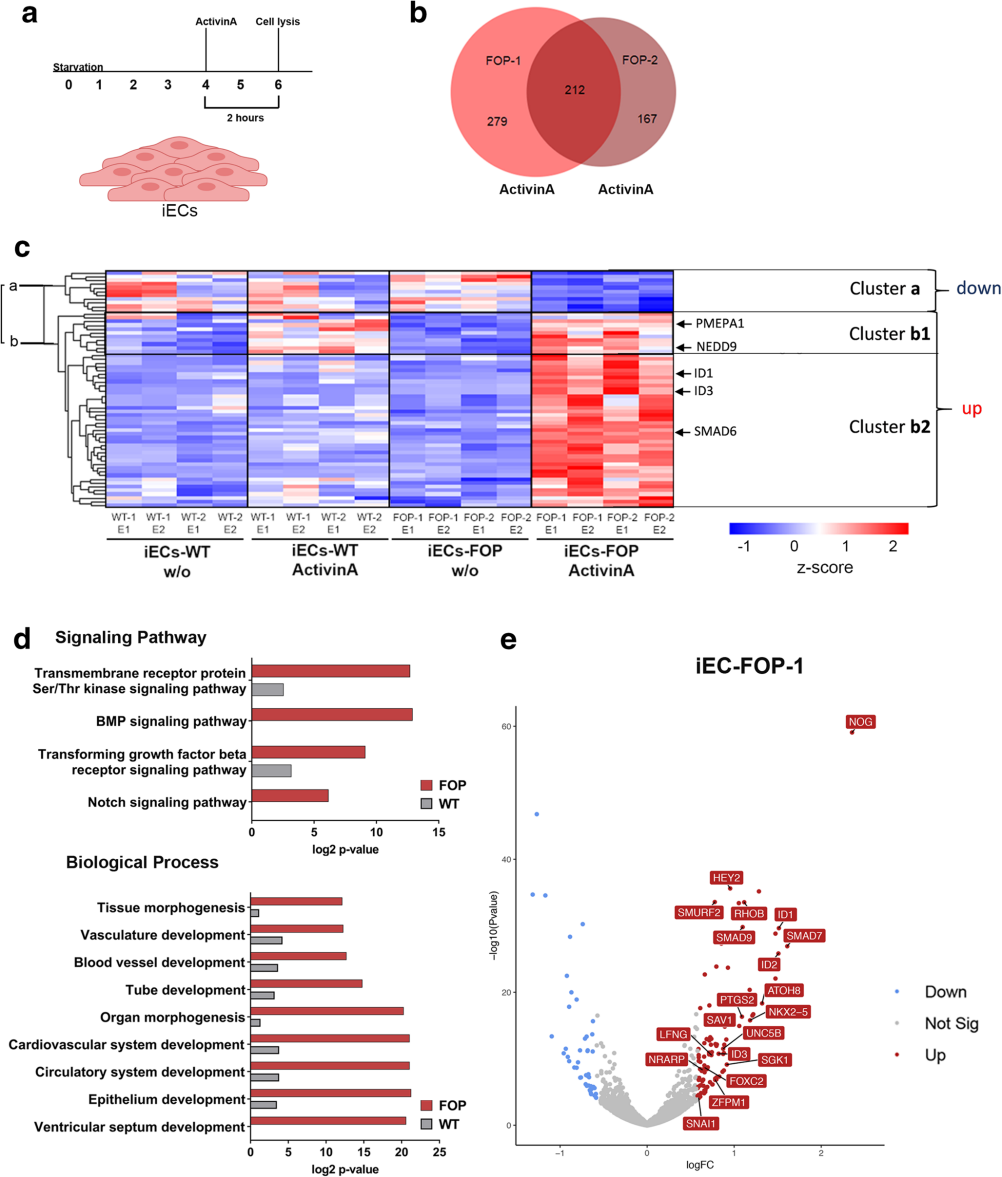
**ActivinA induces a FOP-specific transcriptome in iECs**. (**a**) Experimental setup: RNA Seq of 4 iEC lines, starved for 4 h and stimulated for 2 h with 5 nM ActivinA. (**b**) Venn diagram of RNASeq data presenting the number of DEG in both iEC donors (FOP-1 and FOP-2) stimulated with ActivinA. (**c**) Hierarchical clustering of shared DEG in both FOP donors (adjusted *p* value<0.05; −0.58 ≤ log2FC ≥0.58) of ActivinA treated and untreated (w/o) iECs. Heatmap color coding shows z-score of DEG (red = high; blue = low). Labeling „up”, „down “refers to DEG in FOP iECs upon ActivinA treatment. (**d**) Selected GO terms of shared upregulated genes in ActivinA treated FOP iECs and respective WT values. Depiction of log2 p value with Benjamini correction. Value is 0 if GO term was not identified. (**e**) Volcano Plot of DEG of ActivinA treated FOP-1 iECs. Genes (adjusted p value<0.05; −0.58 ≤ log2FC ≥0.58), up−/downregulation is indicated by color. Genes associated with GO terms are labeled. (DEG; Differentially expressed genes)

We performed whole transcriptome analysis of ActivinA treated WT and FOP iECs via RNA sequencing (RNASeq). Differentially expressed genes (DEG) were analyzed and compared between untreated and ActivinA (2 h) treated iECs from experimental replicates of each donor (Fig. 3a). Two independent FOP donors were stimulated with ActivinA and shared 212 DEG, whereof 64 showed a fold change (FC) of ≥1.5. Those genes were subjected to hierarchical cluster analysis comparing WT and FOP (Fig. 3c). The z-score indicates that most genes in FOP iECs were up- and only few were downregulated by ActivinA (Cluster a, b). In WT iECs, cluster a and b did not show any significant regulation upon ActivinA treatment except of sub-cluster b1, which included the SMAD2/3 target genes *PMEPA1/TMEPAI* and *NEDD9* (Fig. 3c and S3a). Cluster b2 instead included SMAD1/5 target genes (e.g. *ID1, ID3, SMAD6*) (Fig. 3c). This indicates that ActivinA signaling leads to classical SMAD2/3 target gene transcription in WT and FOP iECs, whereas in FOP iECs additional genes were upregulated, including classical BMP/SMAD1/5 target genes. Accordingly, functional gene ontology (GO) annotation revealed significant association between upregulated genes and the BMP pathway and interestingly also the NOTCH pathway only in ActivinA treated FOP iECs (Fig. 3d). Integration of BMP and NOTCH signaling regulate vascular patterning of sprouting blood vessels [32], confirmed here as the GO analysis revealed blood vessel and vascular development in ActivinA treated FOP iECs (Fig. 3d and S3b). GO analysis of upregulated WT genes (FC of ≥1.5) identified TGF-β as the main associated signaling pathway and cell communication as the main biological function (*p* ≤ 0.05) (Fig. 3d and S3d). In summary, ActivinA induced pSMAD1/5 only in FOP iECs resulting in a FOP transcriptome consisting of highly enriched genes (e.g. *ID1, NOG, HEY2, LFNG, UNC5B* (Fig. 3e and S3c)), which are involved in blood vessel formation and activation of BMP and NOTCH pathways.

### ActivinA and BMP6 Upregulate the Same Target Genes in FOP iECs

**Fig. 4:**
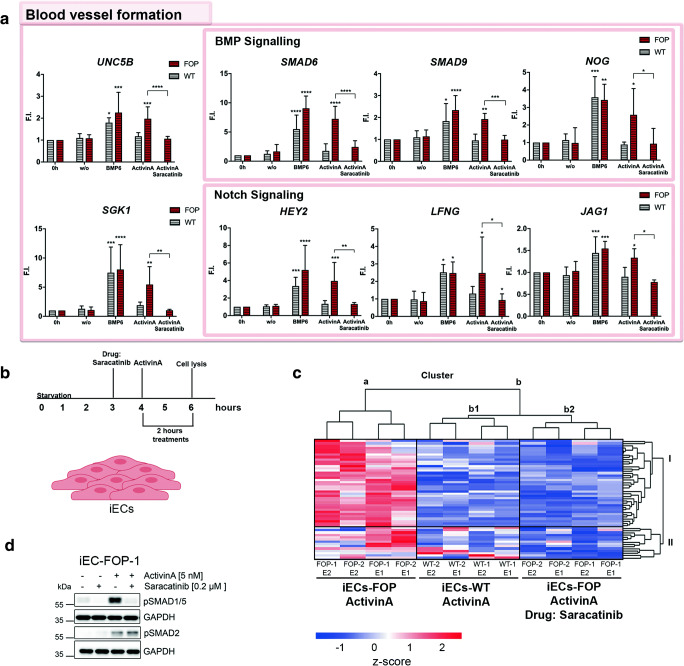
**Saracatinib rescues ActivinA/SMAD1/5 signaling responses in FOP iECs** (**a**) RT-PCR validation of RNASeq target groups upon 1 h pretreatment with Saracatinib (0.2 μM) and 2 h ActivinA (5 nM), BMP6 (5 nM) treatment in iECs. Data is represented as mean F.I. ± SD. (**b**) Experimental setup: RNA Seq of 4 iEC lines, starved for 4 h, pretreated with Saracatinib and stimulated for 2 h with 5 nM ActivinA. (**c**) Independent hierarchical clustering of upregulated genes (adjusted p value<0.05; log2FC ≥0.58) in FOP iECs in comparison to WT and Saracatinib pretreated FOP iECs upon ActivinA (5 nM) treatment. Heatmap color coding shows z-score (red = high; blue = low). (**d**) Representative Western blot of of iECs pretreated with Saracatinib (0.2 μM) and ActivinA (5 nM) (F.I.; fold induction), * p < 0.05, ** p < 0.01, ***p < 0.001, ****p < 0.0001. Significance was calculated relative to unstimulated (w/o) using two-way ANOVA

To further dissect whether ActivinA mediates the same downstream responses as BMP6, we treated cells with BMP6 at the same dose as ActivinA. While BMP6 upregulated the same BMP target genes (*SMAD6, NOG, SMAD9,* ID1/2/3) in WT and FOP iECs, ActivinA led to upregulation of those genes only in FOP (Fig. 4a, 2d and S2e). The same was observed for shared NOTCH target genes and those related to blood vessel formation (Fig. 4a), pointing towards an ActivinA specific mechanism in FOP, absent in WT iECs. Synergistic effects on NOTCH target genes by BMPs in regulating EC specification were reported previously [32]. Several NOTCH target genes, including *LFNG, JAG1, HEY2,* were confirmed as SMAD1/5 targets by chromatin immune precipitation sequencing [33]. Moreover, *UNC5B* and *SGK1* were identified as EC-specific SMAD1/5 targets [33, 34]. However, to our knowledge, this is the first report interlinking ActivinA signaling to NOTCH target gene activation in ECs in the context of FOP.

### Drug Candidate Saracatinib Rescues ActivinA/SMAD1/5 Signaling Responses in FOP iECs

The hypothesis that aberrant and hyperactivated SMAD1/5-dependent signaling of FOP- ACVR1 triggers HO in FOP has advanced the development of several drugs. Here, we used the kinase inhibitor Saracatinib (AZD-0530), a drug candidate for FOP [35] to investigate its inhibitory action in our in vitro disease model.

Saracatinib, initially discovered as a tyrosine kinase inhibitor and developed for the treatment of cancer [36] was later extended as an inhibitor for BMP type I receptors [37] and HO [38, 39]. The effect of Saracatinib on FOP endothelium has not been investigated yet. Here, we focused on early mechanistic actions of Saracatinib on endogenous ACVR1 signaling and its effect on the FOP transcriptome. iECs pretreated with Saracatinib for 1 h (Fig. 4b) followed by ActivinA stimulation inhibited pSMAD1/5 in both FOP donors, whereas pSMAD2 levels remained unaffected (Fig. 4d and S4a). Moreover, independent hierarchical cluster analysis of RNASeq data demonstrated rescue of the transcriptome induced by ActivinA in FOP iECs (Fig. 4c cluster a) to WT level after Saracatinib treatment (Fig. 4c cluster b1 and b2). Of note, Saracatinib preserved ActivinA induced transcription of the SMAD2/3 target genes in WT and FOP iECs (Fig. S3a and 4c cluster II).

## Discussion

Early HO lesions are highly vascularized and FOP patient biopsies uncovered increased vessel number compared to non-hereditary HO [8]. Here, we optimized iPSC differentiation and present the first FOP iEC model, which recapitulates the gain of pathogenic ActivinA/SMAD1/5 signaling.

This is supported by a recent study showing ActivinA/SMAD1/5 signaling in primary ECs isolated from FOP patients [40] indicating that our iECs resemble primary ECs characteristics, providing a valuable, readily available source.

Our results differ from previous iPSC studies, which showed reduced viability [14] and no ActivinA/SMAD1/5 signaling in FOP iECs [15]. We found that FOP iPSCs already show aberrant ActivinA/SMAD1/5 signaling, prompting us to perform mesoderm induction only with exogenous BMP4 (without ActivinA supplementation) [21], while above mentioned studies used established methods for iEC generation (with exogenous ActivinA, BMP4) [41, 42]. BMP4 is essential for mesoderm formation in vivo [43] and in vitro [44], whereas ActivinA was shown to promote mesoderm in vitro but still relies on co-treated BMP4 [45]. Interestingly, different ActivinA, BMP4 doses give rise to multiple mesoderm subsets prior to EC formation in vitro [46], which may be an indication for different EC type generation. Advances in characterization of iEC types, differentiated via distinct routes will advance the understanding of EC heterogeneity in healthy and diseased human tissue.

The underlying mechanism of ActivinA/SMAD1/5 signaling is not fully understood but suggests ACVR1 dependency and independency of ACVR1B/C/TGFBR1 as demonstrated by inhibitor experiments in FOP iECs being in line with knockdown studies in FOP [12] and myeloma cells [47]. Downstream effects of ActivinA/SMAD1/5 signaling in human tissue remain poorly understood. Our study provides first insight of whole genome responses to ActivinA in ECs with upregulation of SMAD2/3 target genes in both WT and FOP iECs and upregulation of additional genes only in FOP iECs. This supports the model that WT-ACVR1 forms a non-signaling complex with ActivinA while binding of ActivinA to FOP-ACVR1 results in an active receptor complex promoting SMAD1/5 signaling [48].

In FOP iECs ActivinA upregulated classical BMP targets like Noggin, a negative feedback regulator that antagonizes certain BMPs but not ActivinA. Additionally, we show ActivinA induced upregulation of NOTCH target genes in FOP iECs. EC-specific disruption of NOTCH signaling in mice impaired bone vessel growth and reduced osteogenesis [49], suggesting that ActivinA could promote coupled angiogenesis and osteogenesis in FOP lesions via NOTCH activation. BMPs synergize or antagonize with NOTCH on different levels to control tip and stalk cell shuffling in blood vessel branching and vascular patterning [1, 32]. We confirmed that ActivinA upregulated the same genes as BMP6 in FOP iECs indicating that mutant ACVR1 lacks ligand specificity by transducing a BMP-like response.

Notably, a model of dynamic pSMAD1/5 regulation in blood vessel development suggests that pSMAD1/5 activates distinct target genes in single ECs thereby pre-patterning the endothelium for tip/stalk cell mediated sprouting [50]. Interestingly, in our study, genes associated with stalk cell identity such as *JAG1, HEY2* were upregulated only in FOP iECs by ActivinA. This is in line with BMP6 induced stalk cell genes via ACVR1 signaling in HUVECs [51].

Therefore, we propose that the ActivinA induced transcriptome via ACVR1/SMAD1/5 signaling pre-patterns the FOP endothelium, which affects tip/stalk cell shuffling in new formed blood vessels in early HO lesions. Potentially, this underlying molecular mechanism contributes to the vascular phenotype found in HO lesions of FOP patients.

However, to functionally recapitulate the complex vascular phenotype in FOP lesions during HO we suggest to integrate iECs in skeletal muscle organoid models [52] to establish a human FOP organoid for future studies. Very recently, fibroblasts were identified as the main source of ActivinA during HO of FOP mice [53]. Fibroblasts proliferate in early pre-osseous HO lesions accompanied by neovascularization [5]. Thus, our iECs can also be used to model human vascularized, fibroproliferative lesions in co-culture experiments with fibroblasts.

Drug testing was performed to rescue the ActivinA induced transcriptome in FOP iECs. The drug candidate Saracatinib prevented only aberrant pSMAD1/5 by ActivinA in FOP iECs, remained SMAD2/3 signaling and successfully restored the FOP transcriptome to WT expression levels. This suggests prevention of aberrant ActivinA effects on the vasculature during HO in FOP patients by the drug candidate Saracatinib and contributes to a better understanding of the specific mechanistic action of Saracatinib in human tissues. Very recently, first clinical investigations of Saracatinib in FOP were initiated (NCT04307953). Thus, we have established iECs as a powerful patient model for further studies on disease mechanism(s) under endogenous receptor levels and for drug testing.

## Supplementary Information

Figure S1 **iPSC and iEC characterization. Related to** Fig. 1**.** (**a**) Sequencing of genomic DNA of iECs from 4 donors at locus of ACVR1 R206H mutation. Arrows indicate point mutation. (**b**) Representative Western blot of lysates from iPSC after stimulation with different doses of ActivinA, BMP6 for 30 min. (**c**) RT-PCR of EC marker in iPSCs compared to iECs. Data is shown as mean normalized expression (MNE) ± SD. * *p* < 0.05, *** *p* < 0.001,****p < 0.0001 using one-way ANOVA. (**d**) Representative phase contrast images of tube-like structures formed by WT and FOP iEs on Matrigel after 24 h. (PNG 2079 kb)

High Resolution Image (TIF 4023 kb)

Figure S2 **ACVR1 signaling characteristics in iECs. Related to** Fig. 2**.** (**a**) RT-PCR of type II receptors in iPSCs compared to iECs. Data is shown as MNE ± SD. (**b**) Relative expression of the TGFβ ligand family shown as FPKM values of RNASeq data of untreated iECs. (**c**) Representative Western blot of lysates from iECs after stimulation with different doses of ActivinA, BMP6 for 30 min. (**d**) or with ActivinA (5 nM) for different time points. (**e**) RT-PCR of BMP target gene *ID3* upon 2 h BMP6 (5 nM), ActivinA (5 nM) treatment in iECs. Data is shown as mean F.I. ± SD. (**f**) (F.I.; fold induction), * p < 0.05,**,** *p* < 0.01,****p < 0.0001. Significance was calculated using one-way (A) and two-way ANOVA (E, relative to unstimulated (w/o)). (PNG 1329 kb)

High Resolution Image (TIF 3429 kb)

Figure S3 **ActivinA downstream signaling responses in iECs. Related to** Fig. 3. (**a**) RT-PCR of SMAD2/3 target genes upon (1 h pretreatment with Saracatinib 0.2 μM) and 2 h ActivinA (5 nM) in iECs. Data is shown as mean F.I. ± SD. (**b**) GO terms of upregulated genes in ActivinA treated FOP iECs. Depiction of log2 *p* value of Benjamini correction (cut-off at adjusted p value<0.01). (**c**) Volcano Plot of differentially expressed genes of ActivinA treated FOP-2 iECs. Genes of (adjusted p value<0.05; −0.58 ≤ log2FC ≥0.58) up−/downregulation is indicated by color. Genes associated with GO terms are labeled. (**d**) GO terms of upregulated genes in ActivinA treated WT iECs. Depiction of all log2 *p* values of Benjamini correction (adjusted p value<0.05). (PNG 1607 kb)

High Resolution Image (TIF 3625 kb)

Figure S4 **Saracatinib rescues ActivinA/SMAD1/5 signaling in FOP iECs. Related to** Fig. 4**.** (**a**) Representative Western blot of protein lysates from iECs pretreated with different concentrations of Saracatinib and stimulation with ActivinA (5 nM) for 30 min. (PNG 213 kb)

High Resolution Image (TIF 1038 kb)
