# Supplementary figures and images for: ActivinA Induced SMAD1/5 Signaling in an iPSC Derived EC Model of Fibrodysplasia Ossificans Progressiva (FOP) Can Be Rescued by the Drug Candidate Saracatinib
Source: Stem Cell Rev Rep. 2021 Jan 7;17(3):1039–52. doi: 10.1007/s12015-020-10103-9 (PMC8166717; doi:10.1007/s12015-020-10103-9)

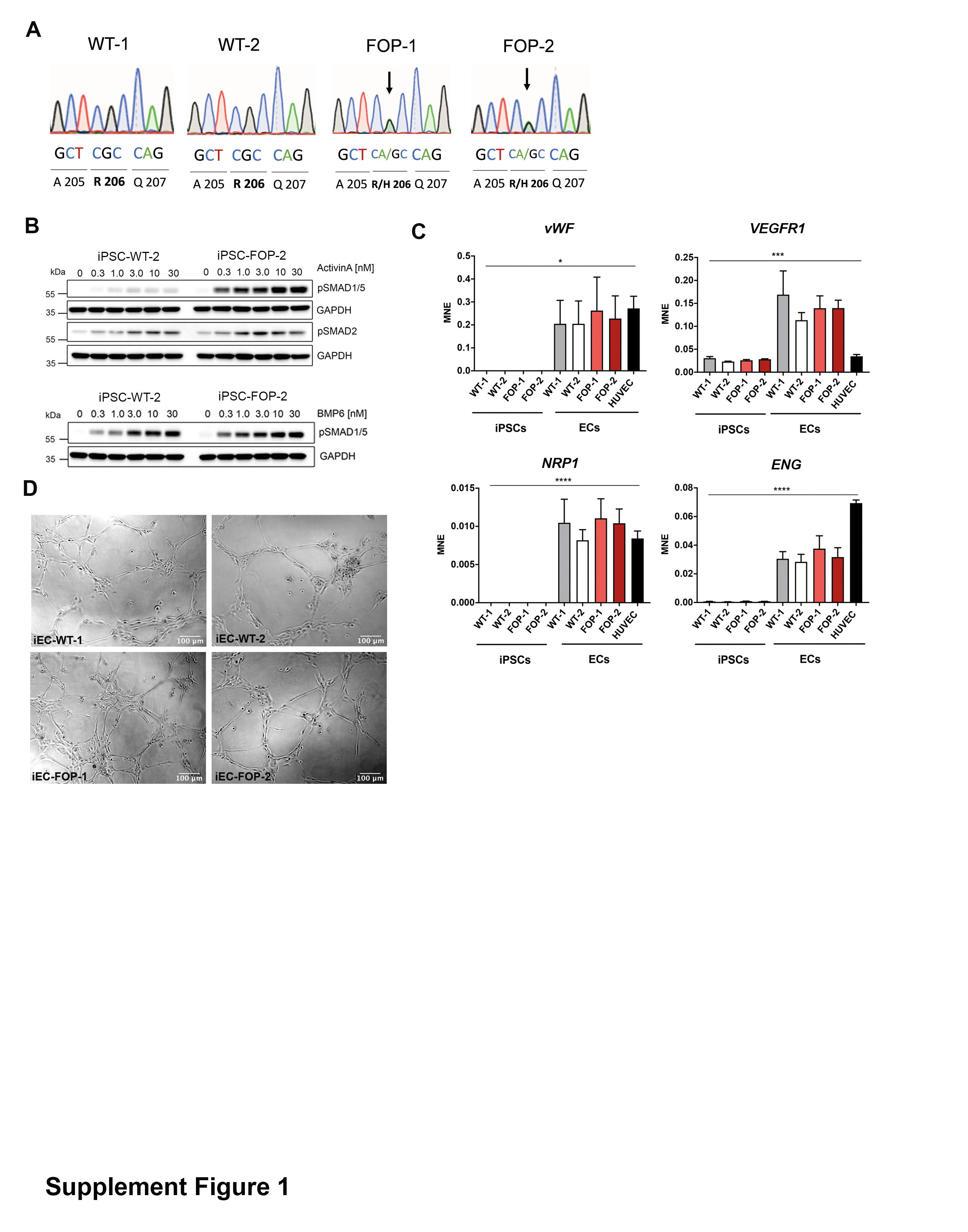

Supplement: Supplementary file 1 — iPSC and iEC characterization. Related to Fig. 1. (a) Sequencing of genomic DNA of iECs from 4 donors at locus of ACVR1 R206H mutation. Arrows indicate point mutation. (b) Representative Western blot of lysates from iPSC after stimulation with different doses of ActivinA, BMP6 for 30 min. (c) RT-PCR of EC marker in iPSCs compared to iECs. Data is shown as mean normalized expression (MNE) ± SD. * p < 0.05, *** p < 0.001,****p < 0.0001 using one-way ANOVA. (d) Representative phase contrast images of tube-like structures formed by WT and FOP iEs on Matrigel after 24 h. (PNG 2079 kb) [file 12015_2020_10103_Fig5_ESM.png]

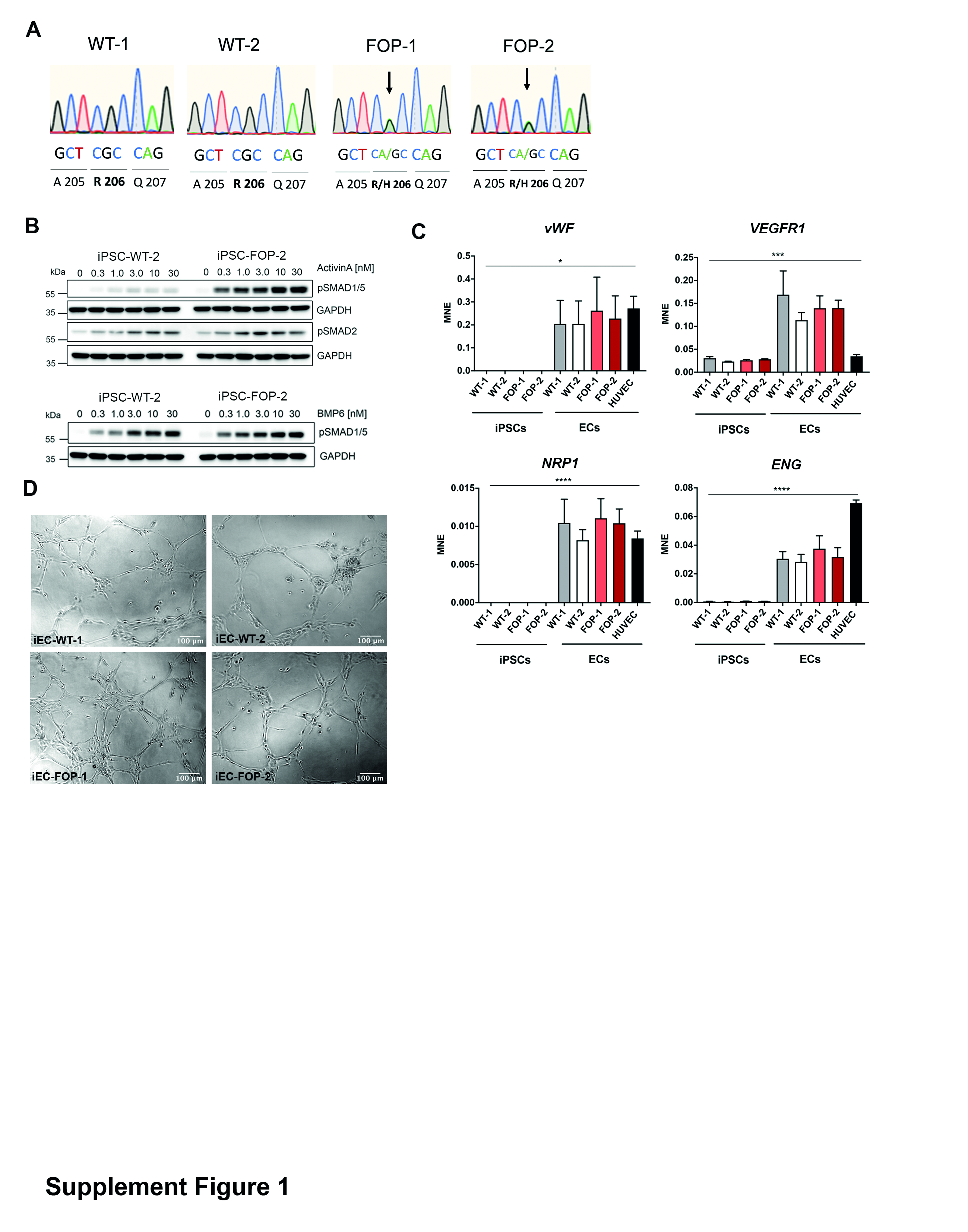

Supplement: Supplementary file 2 — High Resolution Image (TIF 4023 kb) [file 12015_2020_10103_MOESM1_ESM.tif]

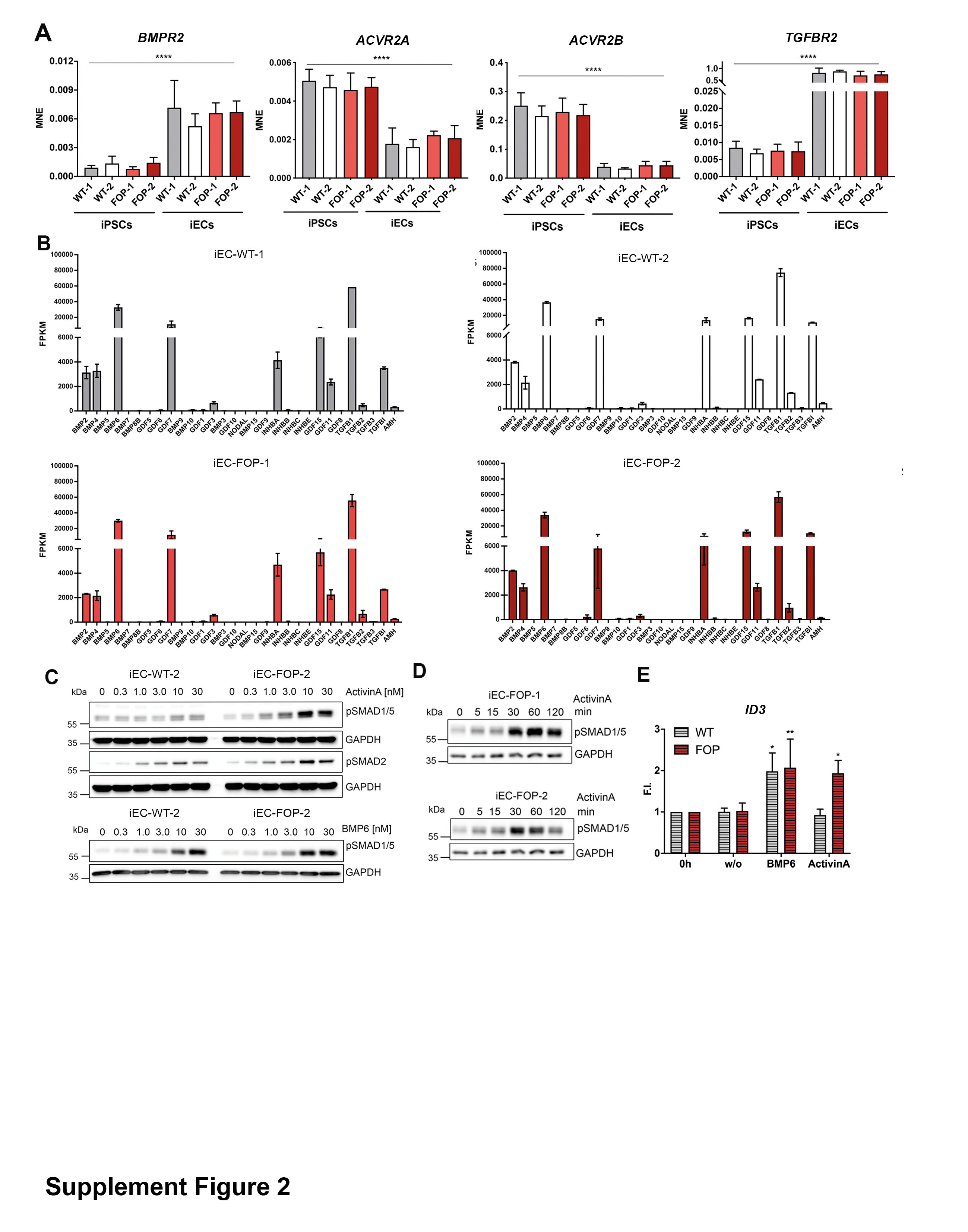

Supplement: Supplementary file 3 — ACVR1 signaling characteristics in iECs. Related to Fig. 2. (a) RT-PCR of type II receptors in iPSCs compared to iECs. Data is shown as MNE ± SD. (b) Relative expression of the TGFβ ligand family shown as FPKM values of RNASeq data of untreated iECs. (c) Representative Western blot of lysates from iECs after stimulation with different doses of ActivinA, BMP6 for 30 min. (d) or with ActivinA (5 nM) for different time points. (e) RT-PCR of BMP target gene ID3 upon 2 h BMP6 (5 nM), ActivinA (5 nM) treatment in iECs. Data is shown as mean F.I. ± SD. (f) (F.I.; fold induction), * p < 0.05,**,** p < 0.01,****p < 0.0001. Significance was calculated using one-way (A) and two-way ANOVA (E, relative to unstimulated (w/o)). (PNG 1329 kb) [file 12015_2020_10103_Fig6_ESM.png]

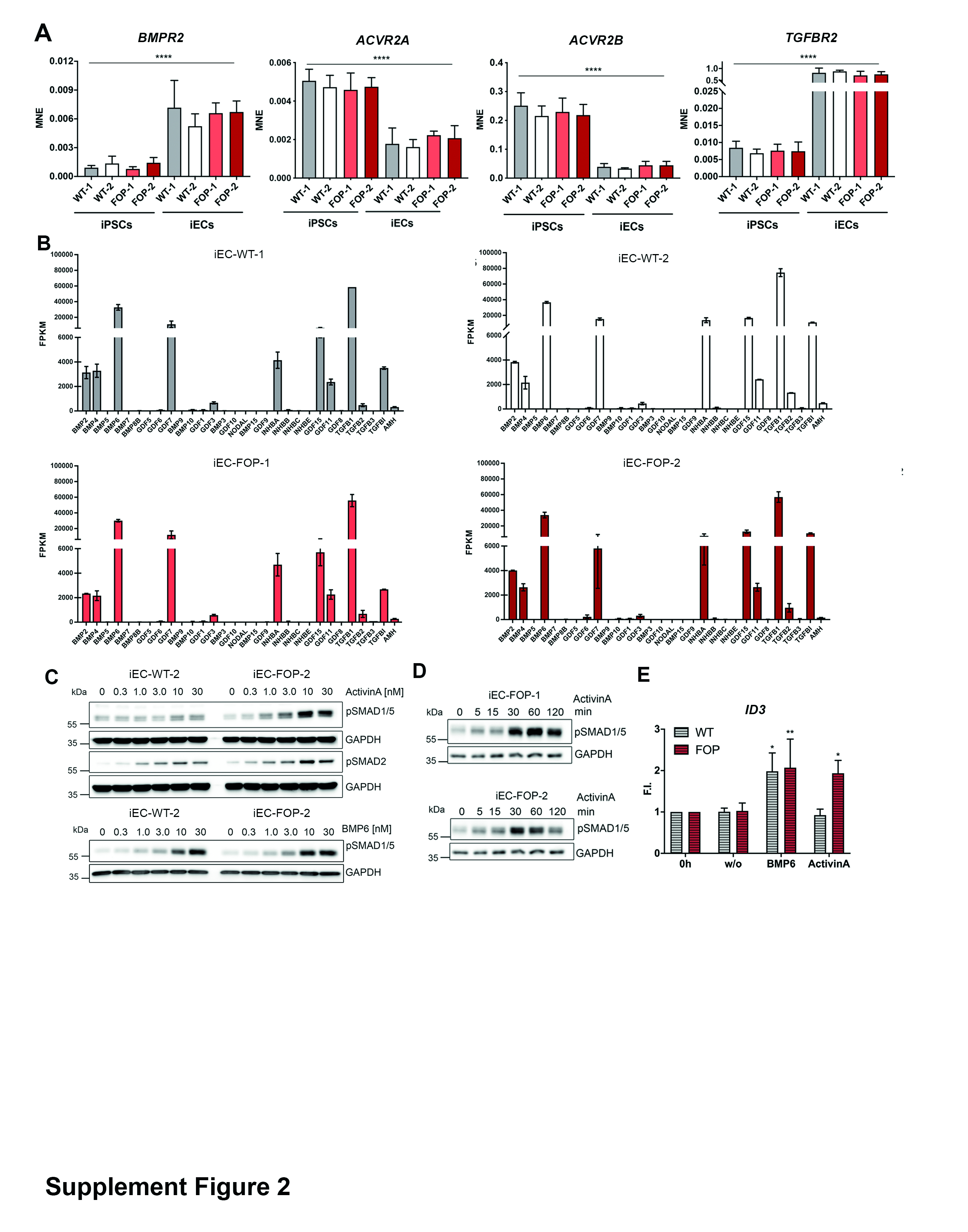

Supplement: Supplementary file 4 — High Resolution Image (TIF 3429 kb) [file 12015_2020_10103_MOESM2_ESM.tif]

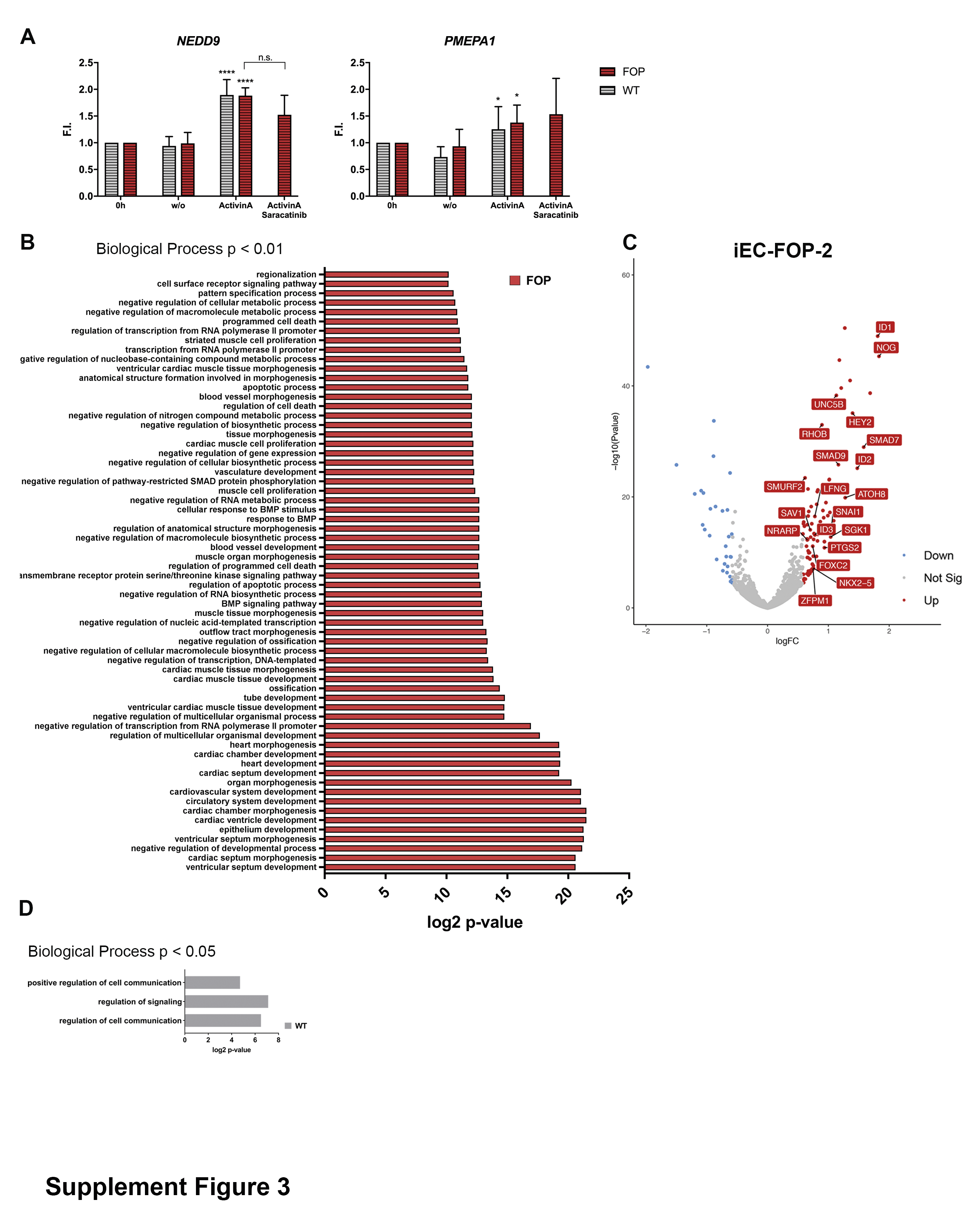

Supplement: Supplementary file 5 — ActivinA downstream signaling responses in iECs. Related to Fig. 3. (a) RT-PCR of SMAD2/3 target genes upon (1 h pretreatment with Saracatinib 0.2 μM) and 2 h ActivinA (5 nM) in iECs. Data is shown as mean F.I. ± SD. (b) GO terms of upregulated genes in ActivinA treated FOP iECs. Depiction of log2 p value of Benjamini correction (cut-off at adjusted p value<0.01). (c) Volcano Plot of differentially expressed genes of ActivinA treated FOP-2 iECs. Genes of (adjusted p value<0.05; −0.58 ≤ log2FC ≥0.58) up−/downregulation is indicated by color. Genes associated with GO terms are labeled. (d) GO terms of upregulated genes in ActivinA treated WT iECs. Depiction of all log2 p values of Benjamini correction (adjusted p value<0.05). (PNG 1607 kb) [file 12015_2020_10103_Fig7_ESM.png]

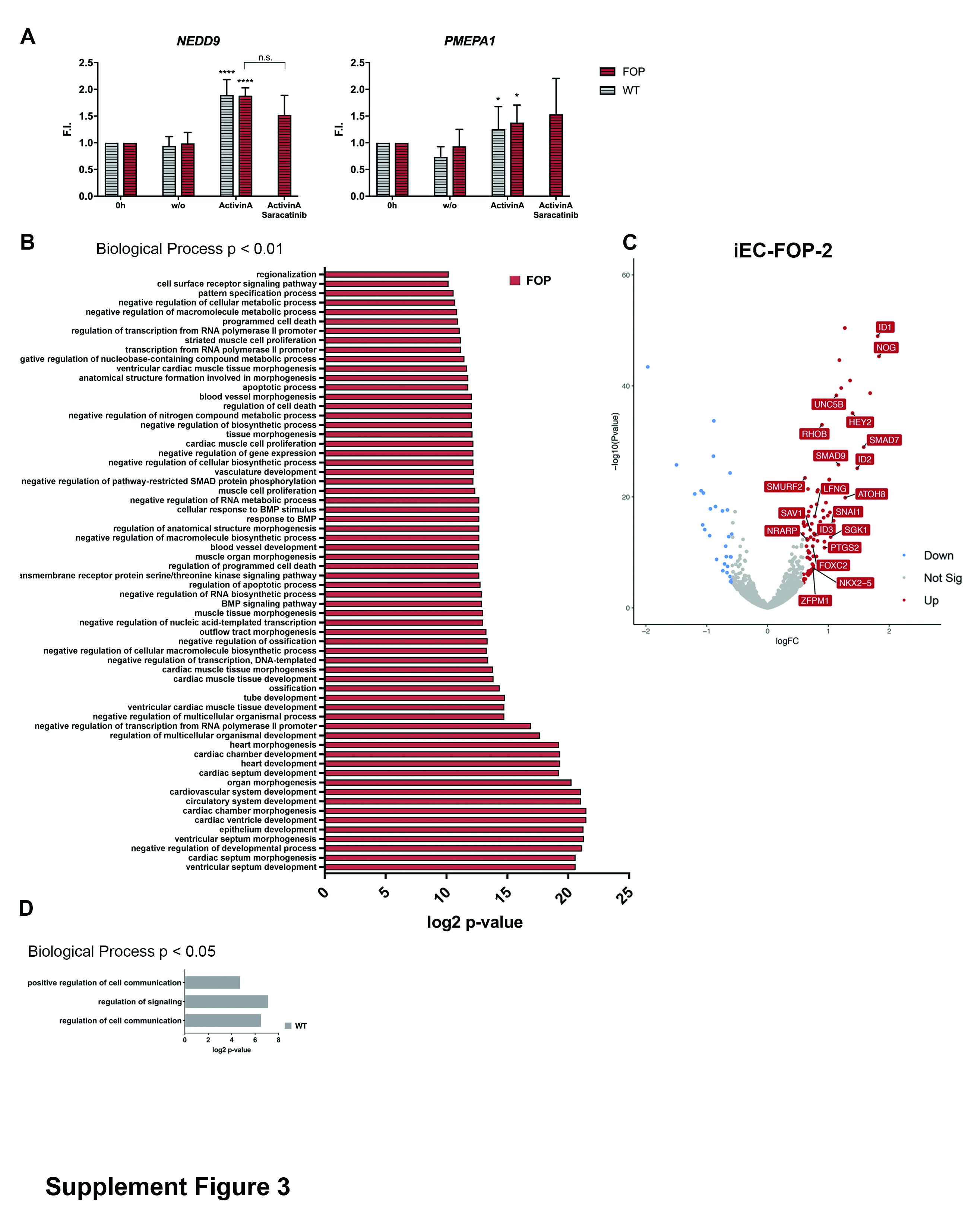

Supplement: Supplementary file 6 — High Resolution Image (TIF 3625 kb) [file 12015_2020_10103_MOESM3_ESM.tif]

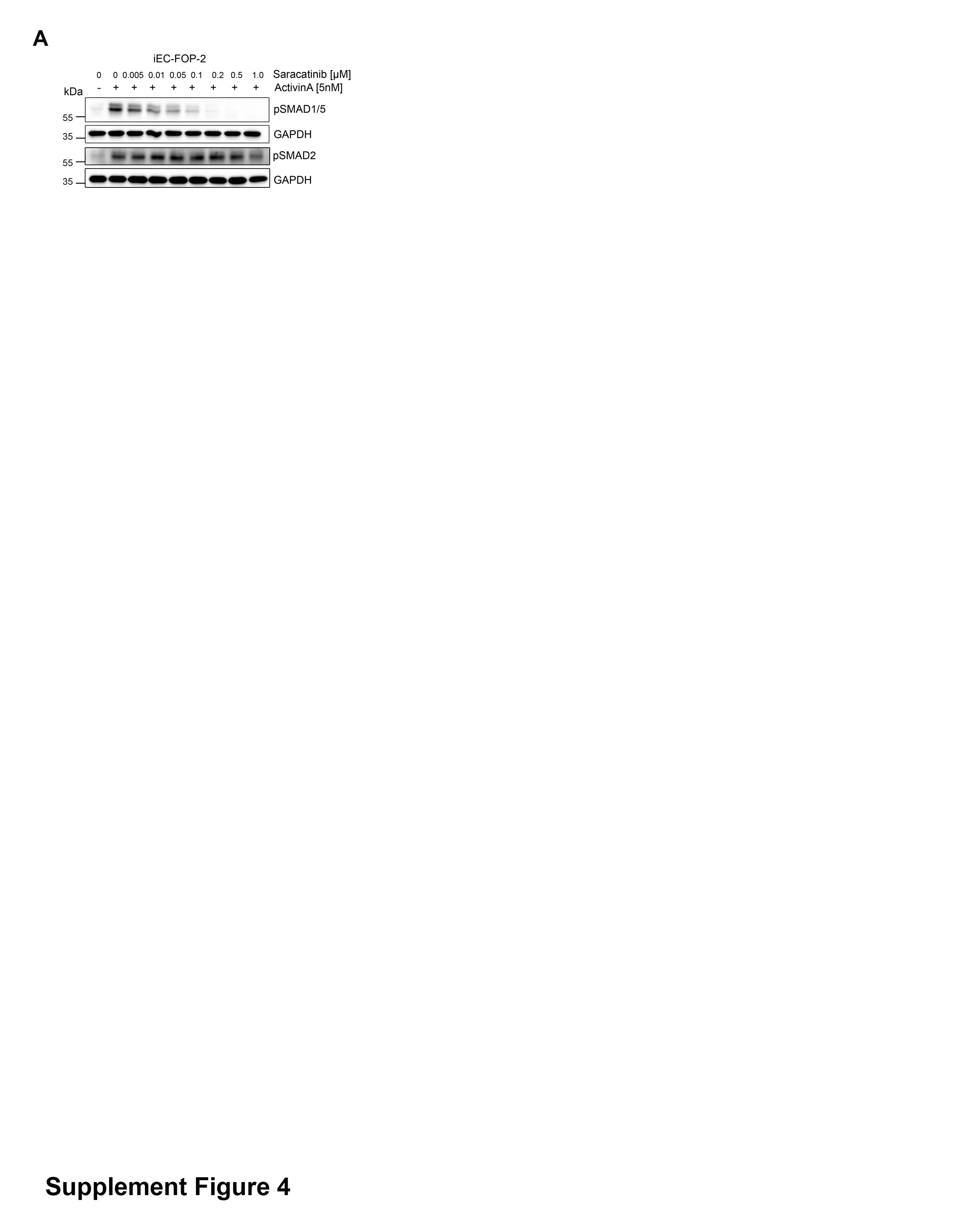

Supplement: Supplementary file 7 — Saracatinib rescues ActivinA/SMAD1/5 signaling in FOP iECs. Related to Fig. 4. (a) Representative Western blot of protein lysates from iECs pretreated with different concentrations of Saracatinib and stimulation with ActivinA (5 nM) for 30 min. (PNG 213 kb) [file 12015_2020_10103_Fig8_ESM.png]

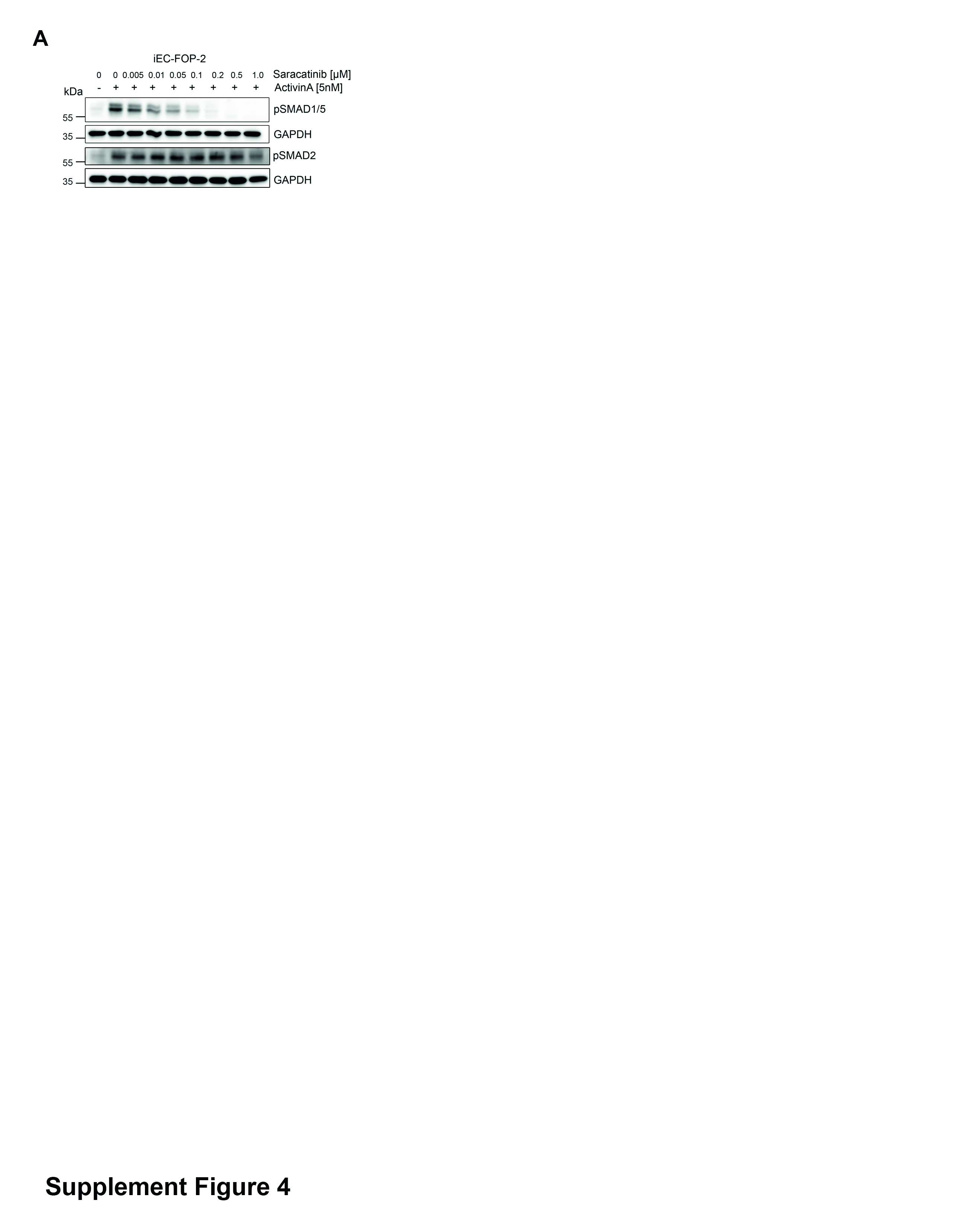

Supplement: Supplementary file 8 — High Resolution Image (TIF 1038 kb) [file 12015_2020_10103_MOESM4_ESM.tif]
